# Supplementary material for: Lipidomic Signature of Healthy Diet Adherence and Its Association with Cardiometabolic Risk in American Adults
Source: Nutrients. 2024 Nov 22;16(23):3995. doi: 10.3390/nu16233995 (PMC11643808; doi:10.3390/nu16233995)
Supplement: Supplementary file 1 [file nutrients-16-03995-s001.zip › nutrients-3285692-supplementary.pdf]

Supplementary Materials

Lipidomic Signature of Healthy Diet Adherence and Its Association with Cardiometabolic Risk in American Adults

Loni Berkowitz[1], Guadalupe Echeverría[1], Cristian Salazar[1], Cristian Faúndez[1], Christopher L. Coe[2], Carol Ryff[2,3], Attilio Rigotti[1].

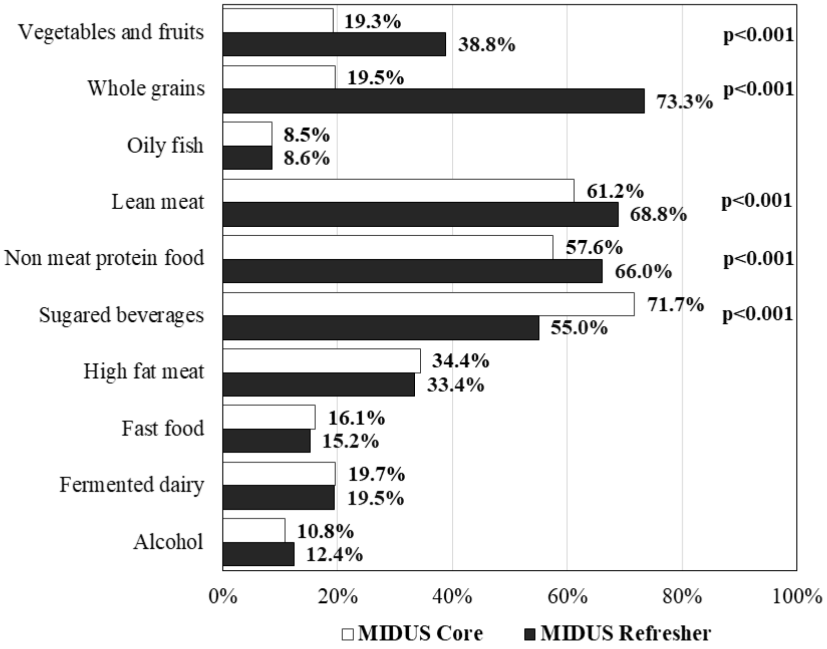

Figure S1. Frequency of maximal score of MIDUS-HEI components by time of sampling in MIDUS study. Chi-squared test was used to compare specific food components between both MIDUS samples.

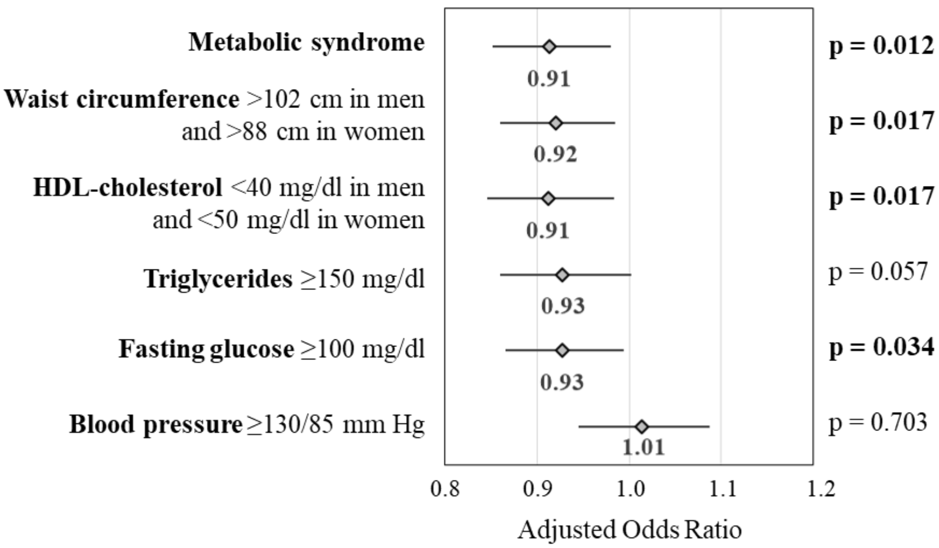

Figure S2. Associations between metabolic syndrome components and diet quality evaluated as MIDUS-HEI. Logistic regression models for the association between metabolic syndrome and each of its diagnostic criteria with diet quality (MIDUS-HEI score) adjusted for time of sampling (MIDUS Core vs. MIDUS Refresher), sex (men vs. women), race (white vs. non-white), age (continuous variable, years-old), educational level (higher education or less and college education vs. postgraduate studies), and smoking status (never, former vs. current smoker).

Table S1. Characteristics of the subsample used to assess cardiovascular risk

**Variables**	**Value in the Subsample (n=1450)**	
Quantitatives	**Mean ± SD**	**Range (min-max)**
Age (years)	57.54 ± 9.8	41 – 79
Education (years)	7.73 ± 2.55	1 – 12
Body mass index (kg/m^2)	30 ± 6.65	14.99 – 65.09
Total cholesterol (mg/dL)	188.82 ± 34	131 – 319
Systolic blood pressure (mmHg)	130.96 ± 17.21	91 – 195
Qualitatives	**Frequency**	**Percentage (%)**
Sex, female	793	54.69
Race, white	1172	80.83
Diabetes mellitus 2, yes	161	11.10
Tobacco, yes	194	13.38
Antihypertensive, yes	377	26.00
